# Applicability of the Chinese Version of the Hypomania Symptom Checklist (HCL-32) Scale for Outpatients of Psychiatric Departments in General Hospitals

**DOI:** 10.1371/journal.pone.0075631

**Published:** 2013-10-07

**Authors:** Xiao Huang, Wenjuan Liu, Bin Feng, Qingrong Tan, Jianlin Ji

**Affiliations:** 1 Department of Psychological Medicine, Zhongshan Hospital, Fudan University, Shanghai, China; 2 Department of Psychiatry, Tongde Hospital of Zhejiang Province, Hangzhou, Zhejiang, China; 3 Department of Psychiatry, Xijing Hospital, Xian, Shanxi, China; Baylor College of Medicine, United States of America

## Abstract

**Objectives:**

This study aimed to determine the suitability of the Chinese version of the Hypomania Symptom Checklist (HCL-32) scale for psychiatric department outpatients with mood disorders in Chinese general hospitals, and provide a theoretical basis for the application of the HCL-32 scale.

**Methods:**

Outpatients with mood disorders receiving continuous treatment in the psychiatric medicine department of three top-ranking general hospitals in three cities completed scoring the HCL-32 scale.

**Results:**

A total of 1010 patients were recruited. 417 were diagnosed with bipolar disorder (236 for type I and 181 for type II) and 593 were depression. Four factors with eigenvalues >1 were considered. Factor 1 with an eigenvalue of 5.5 was labeled “active/cheerful”. Factor 2 with an eigenvalue of 2.7 was labeled “adventurous/irritable.” The coefficient of internal consistency reliability of the HCL-32 total scale was 0.84, and the coefficients for factors 1 and 2 were 0.84 and 0.88, respectively. With the total score of HCL-32≥14 as positive standard, the sensitivity of HCL-32 was calculated at 69.30% and the specificity was 97.81%.

**Conclusions:**

Results showed that HCL-32 had a preferable reliability and validity and was suitable as auxiliary means for bipolar disorder screening in general hospitals.

## Introduction

Bipolar disorder (BD) is a group of diseases caused by various factors, clinically characterized by significant changes in mood or emotion. This disorder is mainly manifested in either elevated or low mood, accompanied by corresponding cognitive and behavioral changes [1]. According to the data on the global burden of disease of the World Health Organization, BD ranks 6^th^ on the list of major disease burdens at the loss of disability adjusted of life year of 2% to 4%, that is, with characteristics of high morbidity, relapse rate, and disease burden [2].

Despite being a serious mental disorder, BD has a relatively low recognition rate even in psychiatric outpatients [3], [4]. The practice guideline for the treatment of patients with BD developed by the American Psychiatric Association indicates that BD II is often misdiagnosed as unipolar depression, thereby preventing patients from receiving proper treatment [5]. Studies have identified factors leading to misdiagnosis of BD and treatment failure: One is that patients in a state of depression generally do not realize that hypomania is a morbidity; consequently, these patients do not inform their clinicians about the condition [6]. Similarly, doctors seldom ask patients with depression about pre-existing hypomania [7]. Consequently, accurate diagnosis and proper treatment become delayed for about 8 to 10 years of the disease course [6], [8]. In addition, studies have indicated that current diagnostic standards are not as reliable and valid as previously thought. These standards include the Diagnostic and Statistical Manual of Mental Disorders 4th Edition (DSM-IV) and The International Statistical Classification of Diseases and Related Health Problems, 10th Revision. Thus, recognition of hypomania needs further investigation. Despite very high specificity, the current diagnostic criteria may have very low sensitivity, focusing more on currently existing symptoms and less on the continuous process of the symptoms. However, monitoring the change in symptoms during the entire course of the disease is essential in the diagnosis of BD [9], [10].

To summarize, the identification of outpatients with BD in its early stages entails difficulty, and missed diagnosis and misdiagnosis have serious consequences. Adoption of a self-rating screening scale significantly helps in the prompt identification of hypomania based on clinical work and epidemiological studies and determination of possible factors that lead to hypomania in patients with depression. The self-rating scale also contributes to BD clinical screening. This study adopted the Chinese version of Hypomania/Mania Symptom Checklist (HCL-32), translated and proofread by experts, to evaluate psychiatric department outpatients with mood disorders in general hospitals in China. Moreover, this study aimed to investigate the validity, sensitivity, and specificity of the Chinese version of HCL-32 applied to Chinese patients with mood disorders and provide a theoretical basis for the application of the aforementioned scale in China by checking the reliability of assessment results.

## Methods

### 1.1. Subjects

The study screened psychiatric department outpatients in three Grade A tertiary general hospitals (Zhongshan Hospital of Fudan University in Shanghai, Xijing Hospital of Xi’an Fourth Military Medical University, and Tongde Hospital of Zhejiang Province) located in three major cities (Shanghai, Xi’an, Hangzhou) in China from November 2009 to December 2011. Inclusion criteria will be as follows: 1) Outpatients in department of psychiatry aged ≥18 years. 2) The patients were diagnosed by psychiatrists to have met the diagnostic criteria of the DSM-IV related to mood disorders. 3) Provision of written, informed consent. Exclusion criteria will be as follows: 1) DSM-IV Disorders except mood disorders. 2) Mood disorders secondary to a general medical or neurological condition. 3) Participation in another clinical study at the same time. Participants signed an informed consent and completed the HCL-32 questionnaires. Another psychiatrist through unified training (attending psychiatrist with more than five years of clinical experiences) used the validated Chinese version of the Mini International Neuropsychiatric Interview (MINI), Version 5.0 to conduct definitive diagnosis and evaluation of patients with depression or BD-I and BD-II [11], [12].

### 1.2. Assessments

The HCL-32 is a self-rating questionnaire that ascertains hypomanic symptoms in patients with MDD [9]. The questionnaire consists of 32 yes/no questions on hypomanic symptoms. Total score is obtained by adding all positive answers. The Chinese version of the HCL-32 has been validated in China. Patients with a total score ≥14 were considered potentially suffering from BD [13].

The diagnostic assessment of BD was conducted using the validated Chinese version of the MINI, Version 5.0 to establish DSM-IV BD-I/BD-II diagnoses [11], [12].

### 1.3. Ethical Considerations

The study was approved by the clinical research ethic committees of zhongshan hospital.

### 1.4. Statistical Analysis

As required by exploratory factor analysis and reliability analysis, we adopted SPSS version 13.0 for data analysis, calculation of the correlation coefficient matrix and the anti-image correlation matrix, conduct of Bartlett’s test of sphericity, and KMO analysis. LISREL 8.50 was used for confirmatory factor analysis.

## Results

### 2.1. Demographic Data

The 1010 outpatients diagnosed with mood disorders (350 from Xijing Hospital Affiliated to Xi’an Fourth Military Medical University, 301 from Tongde Hospital of Zhejiang Province, and 409 from Zhonshan Hospital Affiliated to Fudan University in Shanghai) consisted of 430 males (42.6%) and 580 females (57.4%) aged between 18 and 80 years, with a mean age of (38.4±15.1) years. (See Table 1 for details.).

**Table 1:** General Status of Outpatients with Mood Disorders.

		Total Number (n = 1010)	%
Gender	Male	430	42.6
	Female	580	57.4
Age (Year)	18 to 29	354	35.0
	30 to 49	412	40.8
	≥50	244	24.2
	Mean Age	38.4±15.1	
Marital Status	Single	324	32.1
	Married	612	60.6
	Widowed	28	2.8
	Divorced	34	3.4
	Separated	12	1.2
Literacy	Illiteracy	23	2.3
	Elementary	79	7.8
	Middle	209	20.7
	High/Vocational	323	32.0
	Vocational and above	376	37.2

### 2.2. Diagnostic Analysis of the Patients

A total of 417 patients were diagnosed with BD, and 593 patients were identified as suffering from depression with MINI. The agreement rate between the outpatient doctor diagnosis and the MINI definitive diagnosis was 74.3% (750/1010). The diagnostic accuracy of BD was 56.7% (228/417), the rate of missed diagnosis was 43.3%, and the diagnostic accuracy of depression was 88.0% (522/593).

A total of 553 patients were initially diagnosed with mood disorders, and the agreement rate between the clinical diagnosis and the MINI definitive diagnosis was 68.1% (378/555), including the diagnostic accuracy of BD at 35.3% (73/207), and that of depression at 87.6% (305/348). A total of 455 previously diagnosed patients made subsequent visits (≥2). The agreement rate between this diagnosis and the MINI definitive diagnosis was 81.8% (372/455), including the diagnostic accuracy of BD at 73.8% (155/210) and that of depression at 88.6% (217/245). Outpatients with mood disorders who made a first visit were compared with those who made a subsequent visit. The agreement rate between the clinical diagnosis and the MINI definitive diagnosis were found to increase from 68.1% to 81.8%. The diagnostic rate of BD was also shown to increase from 35.3% to 73.8%. This result, which reflects an increase of 38.5%, is higher by a significant difference of 1.0% than the diagnostic accuracy for depression.

### 2.3. Current Mental Status and HCL-32 Score

Table 2 compares the mean of the HCL-32 total scores among different current mental status No significant difference in the mean of the HCL-32 total scores is indicated (*P* = 0.58), suggesting that the mental status of patients during the visits had no significant effect on the HCL-32 total scores.

**Table 2:** HCL-32 Total Scores of Groups by Current Mental Status.

Current Status	N	HCL-32 Total Scores
Much worse than usual	55	16.4±5.2
Worse than usual	138	15.7±7.8
A little worse than usual	304	16.1±5.6
Neither better nor worse than usual	348	15.2±6.2
A little better than usual	103	15.9±5.9
Better than usual	43	16.8±5.4
Much better than usual	19	16.2±4.6
*P* value (Kruskal–Wallis test)		0.58

### 2.4. Analysis of Each HCL-32 Factor

The matrix formed by the scores corresponding to the questionnaire items was evaluated for its suitability to performfactor analysis. The Kaiser–Meyer–Olkin (KMO) coefficient and Bartlett’s test of sphericity were determined. The KMO coefficient in the present study was 0.74, and Bartlett’s test of sphericity obtained P<0.01, suggesting that the correlation matrix can be used to perform factor analysis. Kaiser’s varimax method was employed for matrix rotation. Consequently, four factors have an eigenvalue >1, with the first two factors having the highest eigenvalues. Factor 1 labeled with “active/elated” had an eigenvalue of 5.5, and Factor 2 labeled with “risk-taking/irritable” had an eigenvalue of 2.7. Factors 1 and 2 constituted 26.2% of the total variation. Based on factor loading of 0.4 for each item, Factor 1 comprised 15 items (Items 2, 3, 4, 5, 10, 11, 12, 13, 15, 16, 19, 20, 22, 24, and 28), whereas Factor 2 comprised 7 items (Items 1, 8, 21, 23, 25, 26, and 27) (See Table 3 for details).

**Table 3:** Analysis of each HCL-32 factor.

Item	N = 1010	
	Factor 1 (Active/Elated)	Factor 2 (Risk-taking/Irritable)
1. I need less sleep	0.34	0.42
2. I feel more energetic and more active	0.62	0.12
3. I feel more self-confident	0.58	0.07
4. I enjoy my work more	0.49	–0.08
5. I am more sociable (make more phone calls, go out more)	0.41	0.24
6. I want to travel and/or do travel more	0.12	0.37
7. I tend to drive faster or take more risks when driving	0.18	0.23
8. I spend more money/too much money	0.31	0.46
9. I take more risks in my daily life (in my work and/or other activities	0.28	0.39
10. I am physically more active (sport etc.)	0.64	0.14
11. I plan more activities or projects	0.70	0.24
12. I have more ideas, I am more creative	0.72	0.11
13. I am less shy or inhibited	0.63	0.18
14. I wear more colourful and more extravagant clothes/make-up	0.23	0.33
15. I want to meet or actually do meet more people	0.58	0.09
16. I am more interested in sex and/or have increased sexual desire	0.40	0.16
17. I am more flirtatious and/or am more sexually active	0.38	0.21
18. I talk more	0.32	0.28
19. I think faster	0.46	0.10
20. I make more jokes or puns when I am talking	0.56	0.12
21. I am more easily distracted	0.23	0.47
22. I am engaged in lots of new things	0.42	0.30
23. My thoughts jump from topic to topic	0.03	0.59
24. I do things more quickly and/or more easily	0.48	0.03
25. I am more impatient and/or get irritable more easily	0.10	0.66
26. I can be exhausting or irritating for others	0.08	0.62
27. I get into more quarrels	0.02	0.57
28. My mood is higher, more optimistic	0.53	0.20
29. I drink more coffee or tea	0.13	0.08
30. I smoke more cigarettes	0.16	0.35
31. I drink more alcohol	0.08	0.21
32. I take more drugs (sedatives, anti-anxiety pills, stimulants)	0.09	0.25

### 2.5. Reliability

The questionnaire uses homogeneity reliability, also known as internal consistency reliability, as the index of reliability test. Cronbach’s alpha coefficient based on item covariance analysis is widely applied in the test of multiple scoring; thus, the coefficient is selected as a homogeneity reliability index. The coefficient of internal consistency reliability of the questionnaire was 0.84, Factors 1 and 2 obtained 0.84 and 0.88, respectively; the lowest homogeneity reliability in all dimensions was 0.63. The homogeneity reliability of the questionnaire was relatively high.

### 2.6. Sensitivity and Specificity of HCL-32

In accordance with the standard of the HCL-32 used to screen for outpatients with mood disorders, a total score ≥14 indicates a positive screen. A total of 289 outpatients with mood disorders based on HCL-32 scores showed a positive screen. In accordance with the MINI definitive diagnosis of BD, 13 showed false positive results, and 128 had false negative results. Sensitivity was calculated at 69.30% (289/417) and specificity, at 97.81% (580/593), as shown in Table 4.

**Table 4:** Sensitivity and Specificity of HCL-32 in General Standard.

Definitive Diagnosis	HCL-32 Positive	HCL-32 Negative
Bipolar disorder	289	128
Non-bipolar disorder	13	580

### 2.7. Comparison between BD and Depression by HCL-32 Evaluation

**Figure 1:**
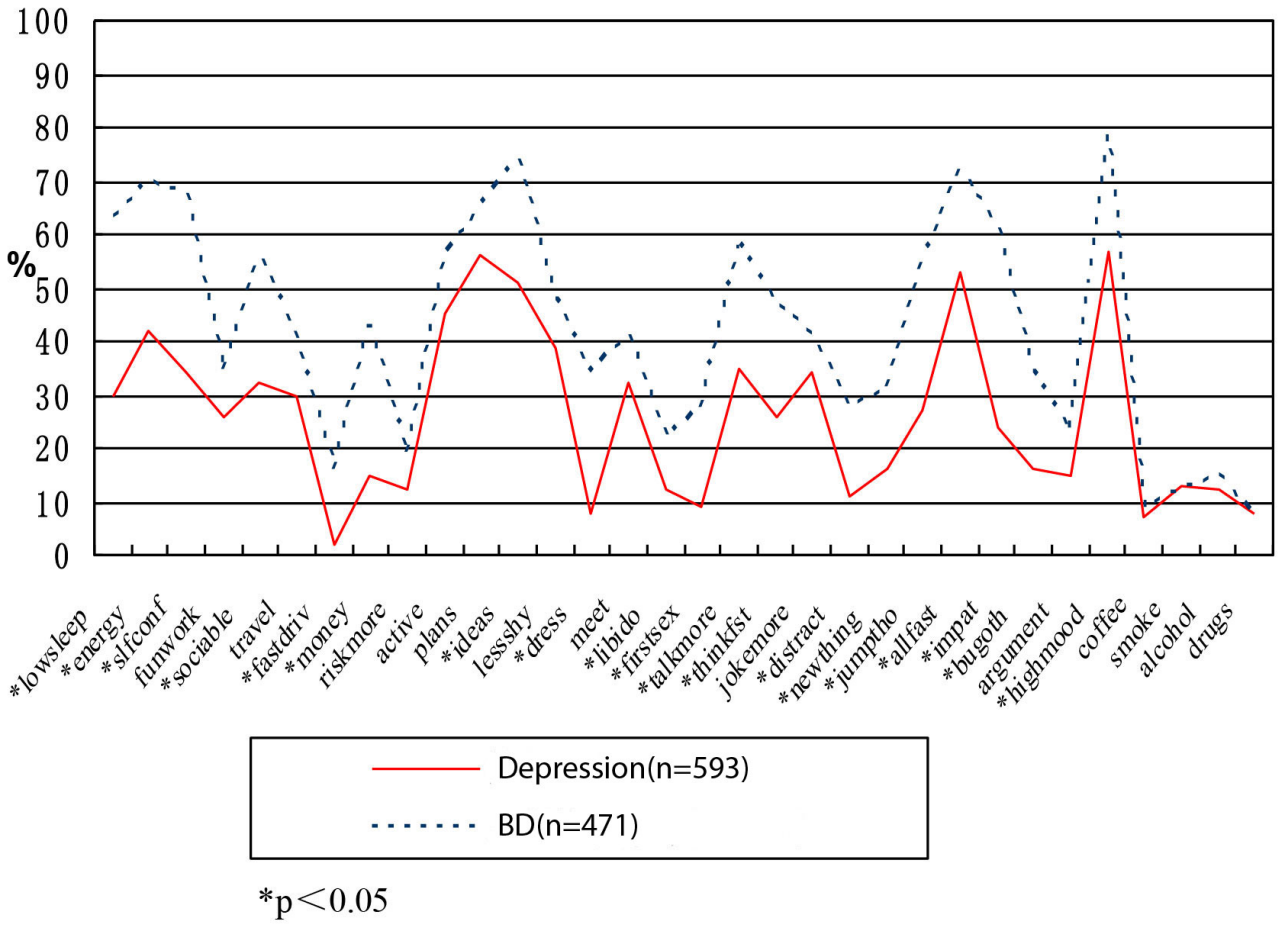
Difference between MDD and BD. Figure shows the analysis and comparison of the item percentages for patients with depression and those with BD by HCL-32 evaluation. Statistical differences were indicated in the evaluation of the 19 items (items 1, 2, 3, 5, 7, 8, 12, 14, 16, 17, 18, 19, 21, 22, 23, 24, 25, 26, and 28).

Table 5 shows the applicability of the HCL-32 for evaluating the percentage of items of all patients in the group with BD and depression, as well as the definitive diagnosis group, false positive group, and false negative group. The two groups are also compared (see Fig. 1 to Fig. 3).

**Figure 2:**
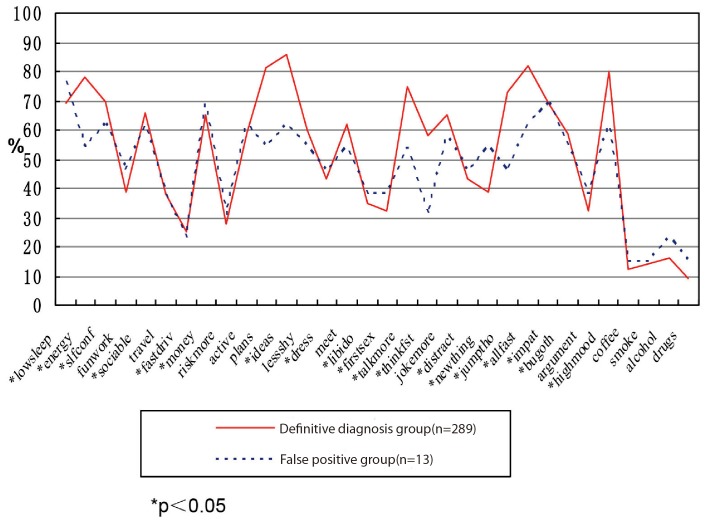
Difference between definitive diagnosis group and false positive group. Figure shows the analysis and comparison of the item percentages for the definitive diagnosis group and false positive group of BD by HCL-32 evaluation. Statistical differences were found in the evaluation of 8 items (items 2, 11, 12, 18, 19, 23, 24, and 28).

**Figure 3:**
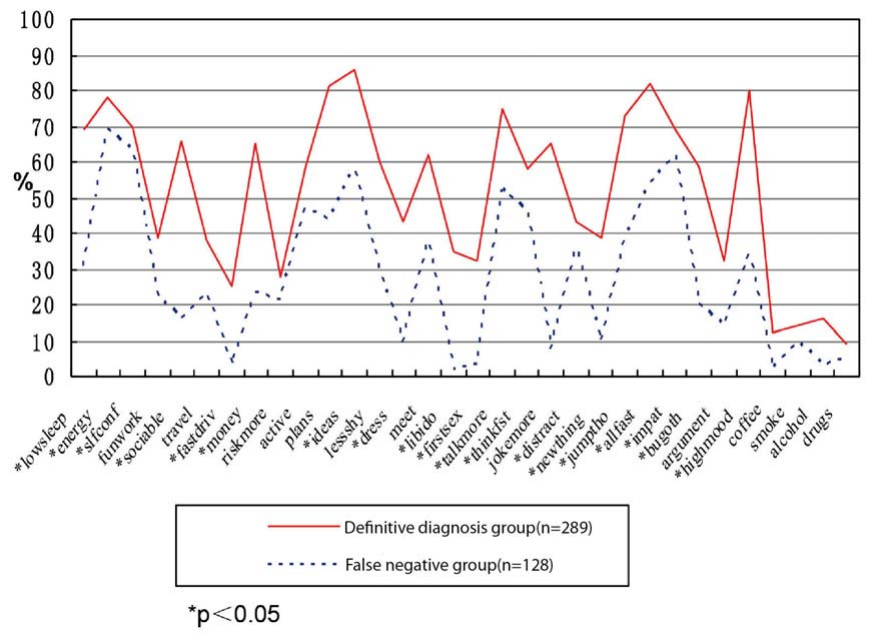
Difference between definitive diagnosis group and false negative group. Figure shows the analysis and comparison of item percentages for the definitive diagnosis group and the false negative group of BD by HCL-32. Statistical differences were found in the evaluation of 23 items (items 1, 4, 5, 6, 7, 8, 11, 12, 13, 14, 15, 16, 17, 18, 20, 22, 23, 24, 26, 27, 28, 29, and 31).

**Table 5:** Overall data for bipolar disorder and depression by HCL-32 evaluation.

Item	Depression(n = 593, %)	BD(n = 417, %)	HCL-32≥14Definitivediagnosis group(n = 289, %)	HCL-32≥14 (n = 13,%) False positivegroup	HCL-32<14 Falsenegative group(n = 128, %)
I need less sleep	30	65	69	77	31
I feel more energetic and more active	42	72	78	54	69
I feel more self-confident	34	70	70	62	63
I enjoy my work more	26	35	39	46	22
I am more sociable (make more phonecalls, go out more)	32	58	66	62	16
I want to travel and/or do travel more	30	41	38	38	23
I tend to drive faster or take more riskswhen driving	2	18	25	23	3
I spend more money/too much money	15	45	65	69	23
I take more risks in my daily life (in my workand/or other activities	12	20	28	31	21
I am physically more active (sport etc.)	45	57	59	62	47
I plan more activities or projects	56	66	81	54	44
I have more ideas, I am more creative	51	76	86	62	58
I am less shy or inhibited	39	49	60	54	29
I wear more colourful and moreextravagant clothes/make-up	8	36	43	46	10
I want to meet or actually do meetmore people	32	42	62	54	38
I am more interested in sex and/or haveincreased sexual desire	12	24	35	38	2
I am more flirtatious and/or am moresexually active	9	30	32	38	3
I talk more	35	60	75	54	52
I think faster	26	49	58	31	46
I make more jokes or puns whenI am talking	34	43	65	58	8
I am more easily distracted	11	30	43	46	36
I am engaged in lots of new things	16	33	39	54	10
My thoughts jump from topic to topic	27	57	73	46	38
I do things more quickly and/or more easily	53	74	82	62	54
I am more impatient and/or get irritablemore easily	24	63	69	69	62
I can be exhausting or irritating for others	16	36	59	54	20
I get into more quarrels	15	24	32	38	14
My mood is higher, more optimistic	57	81	80	62	34
I drink more coffee or tea	7	10	12	15	2
I smoke more cigarettes	13	13	14	15	10
I drink more alcohol	12	16	16	23	3
I take more drugs (sedatives, anti-anxietypills, stimulants)	8	7	9	15	5

## Discussion

The present study involves psychiatric department outpatients with affective disorder in general hospitals. The diagnosis rate of BD was 56.7%. The rate of missed diagnosis reached 43.3%, which was lower than the diagnostic accuracy of 88% for depression. The diagnostic rate of BD was relatively low, as reported by Zhang (2002) [14]. In the Department of Psychological Medicine of Zhongshan Hospital Affiliated to Fudan University in Shanghai, the rate of BD cases was 1.44%, and the rate of missed diagnosis was 42.3%. The findings of this study are consistent with the results of the study by Zhang (2002). In Europe, BD is properly diagnosed in the second or third visit of the patient, who is administered prophylactic treatment. Ghaemi (2002) found that BD usually occurs between the ages of 15 and 20 years; however, BD is often diagnosed between the ages of 25 and 30 years. The misdiagnosis rates of BD were higher than 40%, and the average misdiagnosed time was 7.5 years [15]. Akiskal (2000) [16] suggested that BD-II was the most common clinical manifestation in BD; at least 50% or 70% of patients with clinical depression should be classified as BD-II. When hypomania occurs, patients perceive that they can think faster than before, their self-confidence and self-assessment improve while maintaining their social ability and enthusiasm for work. Therefore, from the perspective of the patients, this emotional state cannot be considered a disease. In addition, in the description of disease history, such emotional experience is often not mentioned in the follow-up clinic unless the clinician inquires persistently. This situation can lead to missed diagnosis, which affects the patient treatment plan as well as the curative effect and prognosis [16].

The present study indicates no statistical difference in the emotional states of patients among the groups after the HCL-32 evaluation. These results are consistent with the research conducted in Italy and Sweden involving patients with mood disorders and another study involving BD-I patients by Yang in China [7], [9], [13]. This similarity suggests that HCL-32 can be used to evaluate patients with mood disorders at any stage and that the results are slightly affected by the state of the disease. The results of HCL-32 evaluation reflect the characteristics of the disease of the patient rather than the current state of performance.

The suitability of the matrix formed by the scores of the questionnaire items to perform factor analysis was evaluated. The KMO coefficient was determined and Barlett’s sphericity test was conducted. The KMO coefficient ranges from 0 to 1. A KMO coefficient above 0.9 is considered highly suitable for factor analysis, between 0.70 and 0.90 is suitable, and below 0.6 is not suitable. When the probability value in Barlett’s test is less than 0.01, the matrix is not considered a unit matrix; thus, factor analysis can be conducted. In this study, the KMO coefficient was 0.71, and Barlett’s test of sphericity obtained a *P* value of less than 0.01. These results indicate that the relevant matrix can perform factor analysis. The result of the HCL-32 factor in this study showed that considering the first 2 factors (factors 1 and 2) yield desirable results. Factor 1 included items 2, 3, 4, 5, 10, 11, 12, 13, 15, 16, 19, 20, 22, 24, and 28, most of which reflected “Active/cheerful” content. Factor 2 included items 1, 8, 21, 23, 25, 26, and 27. Most of which reflected “risk-taking/irritable” content. Factors 1 and 2 were considered in the study conducted among outpatients with mood disorders in Italy and Sweden. Items belonging to these factors were regarded as (active/elated) items–subscale (including item 2, 3, 4, 5, 6, 10, 11, 12, 13, 15, 16, 18, 19, 20, 22, 24 and 28). Factor 2 was regarded as “risk-taking/irritable” item-subscale (including items 7, 8, 9, 21, 23, 25, 26, and 27). These results are similar to those in previous studies [9]. In the study by Yang, the factor analysis of HCL-32 showed that Factors I and II comprised 19 and 6 items, respectively. The results also revealed that the items in Factor II reflected “active/cheerful” content, whereas the items in Factor I mainly involved “risk-take/irritable” and “active/cheerful” contents. This inconsistency could be attributed to the different subjects. The subjects in the study by Yang were those diagnosed with BD-1, who manifested more obvious symptoms such as impulsion and irritability [17].

The questionnaire adopts homogeneity reliability as an index of reliability test. Homogeneity reliability is also called internal consistency reliability. The common computing method includes the bisection method and covariance analysis of the project. Cronbach’s alpha coefficient based on the covariance analysis of the project is suitable for tests of multiple scoring, which is widely used. Thus, the coefficient is selected as the homogeneity reliability index. Cronbach’s alpha coefficient ranges from 0 to 1; if the alpha value increases, the correlation between the questionnaire items increases as well. Generally, if the alpha value exceeds 0.8, the internal consistency is extremely good; between 0.6 and 0.8, the internal consistency is relatively good; and below 0.6, the internal consistency is relatively poor. In the present study, the coefficient of internal consistency reliability of the HCL-32 was 0.84, whereas the coefficients for Factors 1 and 2 were 0.84 and 0.88, respectively. Thus, the questionnaire homogeneity reliability was high and complied with the requirements for psychological measurement and evaluation. The results of this study were very similar to those conducted in Europe [7], [9], which showed internal consistency of 0.82 and 0.86 in the Italian and Swedish samples, respectively. These figures indicate stability in the internal consistency of HCL-32.

A total of 417 patients were definitively diagnosed with BD, of which 289 were positive cases and 128 were negative; the sensitivity was 69.30%. Moreover, 417 cases were definitively diagnosed with depression, of which 13 were positive and 580 were negative; the specificity was 97.81%. Therefore, HCL-32 was used primarily because of its importance. Even in patients with BD of low prevalence rate, relatively high negative predictive validity can be observed. The purpose of the study was to identify possible hypomania factors that could affect patients in the cycle of depression, which is helpful for the clinical diagnosis of BD-II. That is, the probability that patients diagnosed with depression would have negative screening results is very low. Thus, patients can be safely administered antidepressants to reduce risk-inducing mania.

The analysis of the percentage of positive response rate of all items of HCL-32 (see Table 5) revealed that among the items answered by patients diagnosed with BD, those that achieved positive response rates higher than 70% were as follows: item 2 (I feel full of energy or the activity increases), item 3 (I feel more confident), item 12 (I have many thoughts and I am quick-minded), item 24 (I do things more quickly and/or more easily), and item 28 (I am more emotionally wrought up and more positive). Items with positive response rate lower than 20% were the following: item 7 (I like driving fast or ignore risk in the driving), item 29 (I drink more coffee or tea), item 30 (I smoke more), item 31 (I drink more wine), and item 32 (I take more medicine). These results are in agreement with the study by Yang in China (Yang 2008). Some items with low positive response rates may be attributed to the condition in the country and cultural background. For instance, item 7 refers to fast driving, which is related to the low average car ownership per capita in China. Item 29, which refers to drinking more coffee and item 31, which refers to drinking more wine, are related to the reduced rate of drinking wine and coffee compared with the data in foreign countries. The positive response rate for item 32, which refers to taking medicines, reflects the resistance of traditional Chinese folks to taking more medicines. This study also compares the data in HCL-32, such as all items with positive response rates of HCL-32≥14 definitive diagnose bipolar disorder group (n = 289) and HCL-32≥14 false positive group (n = 13) (see Fig. 3). Although the total score of the false positive group is higher than 14, some scores on items showing symptoms of mania were lower than HCL-32≥14 definitive diagnosis bipolar disorder group (*P*<0.05). Among these items were item 2 (I feel full of energy or the activity increases), item 11 (I have many plans of activity or many planned activities), item 12 (I have many thoughts and I am quick-minded), item 19 (My thought is quicker), item 23 (My thought often jumps from one topic to another one) and so on. In addition, 23 items in the HCL-32 questionnaire obtained positive response rates in the false negative group (n = 128) markedly lower than HCL-32≥14 definitive diagnosis BD group (*P<*0.05). However, item 2 (I feel full of energy or the activity increases), item 3 (I feel more confident), item 10 (My activities increase), item 19 (My thought is quicker), item 21 (I divert my attention more easily), item 25 (I am more impatient and/or get angry with others more easily), and other evaluation of core symptoms of mania had no statistical differences when compared with the positive response rate of definitive diagnosis BD group.

The treatment of BD compared with depression is more difficult; the prognosis is worse, and the risk of suicide is higher [18]. Therefore, psychiatric department in general hospitals should not limit its focus on depression but also be on guard against BD, especially BD-II. The HCL-32 has a great clinical value in the department of psychiatry in general hospitals.
